# Active Site Mutations Change the Cleavage Specificity of Neprilysin

**DOI:** 10.1371/journal.pone.0032343

**Published:** 2012-02-23

**Authors:** Travis Sexton, Lisa J. Hitchcook, David W. Rodgers, Luke H. Bradley, Louis B. Hersh

**Affiliations:** 1 Department of Molecular and Cellular Biochemistry, The Center for Structural Biology, University of Kentucky, Lexington, Kentucky, United States of America; 2 Department of Anatomy and Neurobiology, University of Kentucky, Lexington, Kentucky, United States of America; University of Canterbury, New Zealand

## Abstract

Neprilysin (NEP), a member of the M13 subgroup of the zinc-dependent endopeptidase family is a membrane bound peptidase capable of cleaving a variety of physiological peptides. We have generated a series of neprilysin variants containing mutations at either one of two active site residues, Phe^563^ and Ser^546^. Among the mutants studied in detail we observed changes in their activity towards leucine^5^-enkephalin, insulin B chain, and amyloid β_1–40_. For example, NEP^F563I^ displayed an increase in preference towards cleaving leucine^5^-enkephalin relative to insulin B chain, while mutant NEP^S546E^ was less discriminating than neprilysin. Mutants NEP^F563L^ and NEP^S546E^ exhibit different cleavage site preferences than neprilysin with insulin B chain and amyloid ß_1–40_ as substrates. These data indicate that it is possible to alter the cleavage site specificity of neprilysin opening the way for the development of substrate specific or substrate exclusive forms of the enzyme with enhanced therapeutic potential.

## Introduction

Neprilysin (NEP), also known as neutral endopeptidase 24.11, CD10, enkephalinase, and CALLA, is a member of the M13 subgroup of zinc-dependent endopeptidases [1]. NEP was originally discovered in rabbit kidney as a peptidase that cleaves insulin B chain [2]. Subsequent studies showed that NEP is widely expressed throughout mammalian tissues, including the lung, male genital tract, fibroblasts, various epithelia, and at neural synapses in the central nervous system [3]–[5]. The enzyme cleaves a variety of physiological substrates including bombesin-like peptides, amyloid β peptides (Aß), leucine^5^ or methionine^5^-enkephalin, bradykinin, atrial natriuretic factor (ANF), and substance P [6]–[9]. NEP exhibits a preference for cleavage on the amino terminal side of hydrophobic residues [10].

Because of its multiple targets, NEP has been the focus of numerous studies attempting to modulate its activity for therapeutic purposes. One such target is the use of NEP to reduce Aß peptide levels in Alzheimer's disease, since the oligomerization of Aß has been linked to the etiology of this disease [11]. Indeed, in studies with transgenic mice NEP expression decreases the level of Aß [12]–[15] and ameliorates cognitive deficits typically attributed to AD [16]. In yet another application inhibitors of NEP were developed to block its “enkephalinase” activity to increase the concentration of enkephalins in the brain and thus their analgesic effect [17]. Peripherally expressed NEP may have a role in appetite control and obesity. NEP deficient mice become obese [18], while a peripherally administered NEP inhibitor that does not cross the blood-brain barrier increased food intake and subsequently led to obesity. Recently, an NEP inhibitor was shown to increase female genitalia blood flow in rabbits by preventing vasoactive intestinal peptide (VIP) cleavage [19]. This could potentially lead to the use of NEP as a therapeutic agent in the treatment of female sexual arousal disorder.

While methods to modulate NEP activity have displayed the potential for therapeutic use, they also reveal a paradox to their usage. For example, using NEP to lower Aß may indeed decrease the amount of the target substrate; it may also have undesired consequences by removing other physiologically important products such as the enkephalins or vasopressin. Alternatively, inhibiting NEP to enhance opioid levels will likely cause an increase in Aß, which would result in an increased risk in the development of Alzheimer's disease.

A strategy to bypass the potential problems associated with the substrate promiscuity of NEP is to alter its specificity towards a target substrate thus reducing potential off-target effects. There is ample precedence to apply such a strategy. For example, substitutions within the active site of trypsin, although decreasing activity, shifted the relative preference for arginine versus lysine [20]. Similarly, a series of mutations in Rous sarcoma virus protease displayed altered amino acid preferences at particular substrate positions, allowing position-by-position control of substrate specificity [21]. Using thermolysin as a homology model, we were able to show that conversion of Val^573^ to Leu produced a form of NEP which reacted with substrates with small P′1 residues essentially the same as wild-type enzyme, yet substrates containing bulky P′1 residues exhibited a decreased V_max_ with little change in K_m_ [22]. This study, although limited in scope, demonstrated the feasibility of altering NEP substrate specificity. The nomenclature of Schecter and Berger (Schechter I, Berger A. (1968) Biochem. Biophys. Res. Commun. 32: 898–902) is used where residues of the substrate C-terminal to the site of cleavage are designated P1′, P2′, P3′, etc as they move away from the scissile bond and residues N-terminal to the scissile bond are designated P1, P2, P3, etc as they move away from the scissile bond. The corresponding binding sites on the enzyme are designated S1′, S2′, S3′, and S1, S2, S3, etc. respectively.

By analyzing the crystal structure of NEP in complex with the inhibitor phosphoramidon [23], we have initiated a rational design approach to mutate NEP active site targeting residues likely to interact with substrates. In this study, we explore NEP substrate specificity by generating NEP mutant libraries of two active site residues, Phe^563^ which is part of the S1′ binding site and Ser^546^ which appears to contribute to the S2/S3 binding site. A number of these mutants displayed differential changes in activity toward physiological substrates including changes in cleavage site preferences. Together, these data support the hypothesis that amino acid changes in the active site of NEP can potentially give rise to therapeutically relevant forms of NEP.

## Results and Discussion

### Selection of sites for mutagenesis

Mutations were made at the Phe^563^ and Ser^546^ sites in a secreted form of human NEP (shNEP) expressed as a C-terminal hexahistidine fusion protein. The NEP crystal structure reveals that Phe^563^ forms part of the S1′ substrate binding pocket believed to impart the preference for hydrophobic/aromatic P1′ residues at this position, **Figure S1**. Phe^563^ is located in a coil region just prior to the helix containing the active site residues. Ser^546^ is part of a ß-sheet lining the substrate-binding site [23] and is positioned to interact with the P2 or P3 residues of a bound substrate on the carboxyl side of the scissile bond. Based on the NEP crystal structure, the position of both Phe^563^ and Ser^546^, and their conservation among species, we hypothesized that these residues contribute to substrate specificity.

### Expression of mutant NEP

To test the contribution of Phe^563^ and Ser^546^ to catalysis we used degenerate oligonucleotides to construct NEP libraries in which we introduced amino acid substitutions at these positions. Substitutions made at Phe^563^ included valine, leucine, methionine, isoleucine, serine, histidine, aspartic acid, arginine, glutamine, asparagine, and lysine. Substitutions made at Ser^546^ included glutamate, lysine, threonine, glycine, arginine, and alanine. Individual mutant library members were transfected in HEK293 cells and analyzed for expression. Of the seventeen sequences examined, five mutants, NEP^F563L^, NEP^F563V^, NEP^F563M^, NEP^F563I^, and NEP^S546E^ expressed at levels near to that of wild-type NEP and were selected for further purification and analysis. The low expression of other mutants appeared to be due to their cellular instability as they all produced similar amounts of mRNA, **Figure S2**, which did not correlate with protein expression nor did the poorly expressing mutants accumulate intracellularly. These results suggest that active-site residues Phe^563^ and Ser^546^ play a role in overall protein folding and/or stability and that the non-expressing mutants were likely degraded intracellularly.

The five expressing NEP mutants were purified by nickel affinity chromatography, and the amount of NEP present determined by Sypro ruby staining of SDS-PAGE gels. We initially compared Sypro Ruby and Western blot analysis for enzyme quantitation and obtained equivalent results with either method, **Figure S3**.

### Reaction of NEP mutants with the synthetic substrate Glut-Ala-Ala-Phe-MNA

Activity assays were first performed using the synthetic peptide Glut-Ala-Ala-Phe-MNA. This substrate is cleaved between the Ala-Phe peptide bond and thus any effects of mutations on the cleavage at this site will be reflected in the reaction kinetics. We demonstrated that Glut-Ala-Ala-Phe-MNA hydrolysis by NEP and each of the studied mutants was completely inhibited by the relatively specific inhibitor, phosphoramidon at 100 µM, and the highly specific inhibitor CGS 24592 [24] at 10 nM, thus demonstrating that hydrolysis was attributed to NEP or its variant and not a contaminating protein.

Kinetic constants for mutants determined with Glut-Ala-Ala-Phe-MNA as substrate are presented in **Table 1**. These kinetic constants were derived under first-order assay conditions monitored in a continuous mode. The wild-type enzyme and the NEP^F563L^ mutant exhibited essentially the same specific activity of 46 and 44 pmoles/min/ng, respectively, while the NEP^F563I^, NEP^F563V^, NEP^F563M^, and NEP^S546E^ mutant activities varied from ∼25% to 45% of the wild-type enzyme, **Table 1**. K_m_ values varied ∼2.5 fold ranging from 51 to 118 µM, with V_max_/K_m_ values varying three fold or less. Thus mutating Phe^563^ and Ser^546^ produce small but detectable affects on the cleavage of Glut-Ala-Ala-Phe-MNA confirming that these residues contribute to catalysis.

**Table 1:** The specific activity towards glutaryl-Ala-Ala-Phe-MNA cleavage is reduced in NEP mutants.

	Specific Activity	K_m_	K_i insulin B chain_
	(pmoles/min/ng)	(µM)	(µM)
NEP	45.9±2.5	51±11	1.9±0.2
NEP^F563I^	17.7±0.6	83±4	2.5±0.9
NEP^F563L^	43.6±0.1	81±11	1.5±0.3
NEP^F563M^	19.2±0.4	87±8	1.2±0.1
NEP^F563V^	11.2±0.3	51±10	1.0±0.1
NEP^S546E^	20.1±0.6	74±10	ND
NEP^S546A^	ND*	118±7	ND
NEP^S546T^	ND*	73±6	ND

All assays were conducted at 37°C at in 20 mM MES buffer, pH 6.5.

ND = not determined.

*The concentrations of NEP^S546A^ and NEP^S546T^ were too low to quantify, however, they were active enough to determine K_m_.

### Reaction of NEP mutants with physiological substrates

We extended the comparison of the various mutants by studying their reaction with three physiological substrates; leucine^5^-enkephalin (leu-ENK = Tyr-Gly-Gly-Phe-Leu), which is cleaved by NEP at the Gly-Phe bond, insulin B chain, which has multiple cleavage sites, and amyloid beta peptide 1–40 (Aß_1–40_), which also has multiple cleavage sites. The rate of cleavage of these peptides was determined by following the disappearance of the parent peptide by reverse-phase high performance liquid chromatography (HPLC). Leu-ENK was cleaved at the Gly-Phe bond by all of the mutants, with rates varying from approximately 80% of the wild-type NEP rate (NEP^F563L^) to less than 20% of the wild-type rate (NEP^F563V^), **Table 2**.

**Table 2:** Rates of hydrolysis of physiological peptides by NEP mutants.

	leu-ENK	Insulin B Chain	Aß_1–40_
	pmole/min/ng	pmole/min/ng	fmole/min/ng
NEP	1.90±0.23	0.86±0.13	198±12
NEP^F563L^	1.57±0.32	1.17±0.18	116±4
NEP^F563M^	1.21±0.22	0.21±0.01	85±3
NEP^S546E^	1.12±0.12	0.58±0.07	93±8
NEP^F563I^	1.06±0.17	0.13±0.02	43±1
NEP^F563V^	0.31±0.02	0.06±0.02	14±1

Hydrolysis was carried out at 37°C at in 20 mM MES buffer, pH 6.5. Substrate concentrations were 15 µM insulin B chain, 24 µM Aß_1–40_, and 64 µM leu-ENK. Activity was determined by following the disappearance of substrate by HPLC. Each reaction was run in at least triplicate. Statistical analysis was conducted using a two-tailed paired t-test with Prism4 software.

The specific activities of the various mutants with the physiological substrate leu-ENK showed a similar pattern as observed with Glut-Ala-Ala-Phe-MNA. NEP^F563L^ had near wild-type levels of activity for both Glut-Ala-Ala-Phe-MNA and leu-ENK, while NEP^F563M^, NEP^F563I^, and NEP^S546E^ all showed approximately 40–60% activity towards these substrates, and NEP^F563V^ exhibited a 75–85% decrease in activity towards both. Thus the effects of these mutations can be attributed to those produced for cleaving N-terminal to single phenylalanine residue.

Insulin B chain and Aß_1–40_ contain multiple cleavage sites. Cleavage at any one of these sites will result in peptide disappearance as determined by HPLC. Compared to wild-type NEP, mutants NEP^F563V^, NEP^S546E^, NEP^F563M^, and NEP^F563I^ all exhibited reduced hydrolysis rates for insulin B chain (p values of 0.03 or lower), whereas with NEP^F563L^ the hydrolysis rate was higher (p = 0.03). With Aß_1–40_ as substrate all of the mutants showed reduced rates of hydrolysis (all exhibited p values<0.05).

When the relative cleavage rates for insulin B chain, Aß_1–40_, and leu-ENK were compared between NEP and the various mutants there was a discernable change in substrate preference. For example, NEP^F563L^ hydrolyzed insulin B chain at a rate 1.4 times faster than NEP, but cleaved Aß_1–40_ at nearly half the rate of the wild-type enzyme. Thus NEP^F563L^ exhibits a greater than 2 fold preference for insulin B chain over Aß_1–40_. NEP^F563V^ cleaved leu-ENK at 1/6 the rate of the wild-type enzyme and Aß_1–40_ at 1/12 the wild type rate, **Table 2**, increasing the preference of this mutant for leu-ENK by two fold. Thus different mutations differentially altered NEP specificity.

To further demonstrate that the NEP mutants differ in their ability to discriminate between substrates, we measured rates of cleavage of a mixture of two substrates, leu-ENK and insulin B chain. The rate of cleavage of a substrate mixture is determined by both the k_cat_ for that substrate as well by the affinity for the substrate. As can be seen in **Table 3** all NEP mutants showed a decrease in the rate of hydrolysis of both leu-ENK and insulin B chain when present together. This is expected, given that each peptide acts as a competitive inhibitor of the other. NEP^F563V^, NEP^F563L^, and NEP^F563M^ all showed approximately the same wild-type enzyme ratio of rates for leu-ENK/insulin B chain indicating no change in overall substrate preference. However, NEP^F563I^ exhibited a shift in substrate preference toward leu-ENK, while the substrate preference for NEP^S546E^ shifted more toward insulin B chain, even though the rate remained higher for leu-ENK. That is the ratio for leu-ENK/insulin B chain cleavage is 3 for NEP^S546E^ compared to 6 for wild-type NEP. Thus these single amino acid substitutions had a measureable effect on substrate preference.

**Table 3:** Leu-Enkephalin and Insulin B chain dual substrate assays.

Mutant NEP	leu-ENK	Insulin B Chain	Ratio leu-ENK insulin B chain
	pmole/min/ng	pmole/min/ng	
NEP	1.16±0.06 (0.61)	0.20±0.04 (0.23)	5.8
NEP^F563V^	0.23±0.03 (0.74)	0.04±0.01 (0.67)	5.8
NEP^F563L^	0.97±0.06 (0.62)	0.19±0.03 (0.20)	5.1
NEP^F563M^	0.69±0.11 (0.57)	0.11±0.02 (0.52)	6.3
NEP^F563I^	0.62±0.03 (0.58)	0.02±0.01 (0.15)	31.0
NEP^S546E^	0.76±0.14 (0.68)	0.25±0.01 (0.43)	3.0

The numbers in parenthesis indicate activity relative to the uninhibited values.

Reactions were carried out at 37°C at in 20 mM MES buffer, pH 6.5 containing 15 µM insulin B chain and 64 µM leu-ENK. Activity was measured and statistical analysis carried out as in **Table 2**.

### Identification of NEP cleavage sites in insulin B chain

We next looked in more detail on the effect of the NEP^F563^ and NEP^S546^ mutations on the hydrolysis rates at individual cleavage sites in insulin B chain. We first tested for a change in the affinity of insulin B chain for mutant NEPs by using insulin B chain as an alternate substrate (competitive) inhibitor of Glut-Ala-Ala-Phe-MNA hydrolysis. We found that there was no dramatic change in the K_i_ for any of the mutants, with variations of two fold or less (**Table 1**).

**Figure 1:**
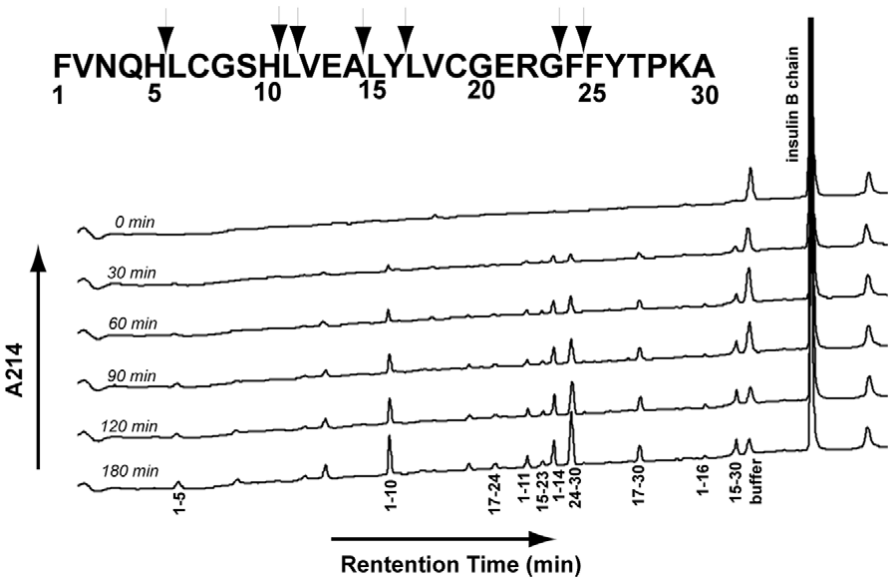
Time course for NEP mediated hydrolysis of insulin B chain. Time course assays were conducted by incubation of NEP with 15 µM insulin B chain in 20 mM MES buffer, pH 6.5, at 37°C. A 100 µL aliquot was removed at each time point and 10 µL of 5% trifluoroacetic acid (TFA) was added to stop further hydrolysis. 95 µL were injected into a Vydac C4 column and developed as described in Materials and Methods. Each product was isolated and subjected to mass spectral analysis. Numbers under each peak indicate the identification of the peptide by sequence. Peaks without numbers were not identified.

Degradation of insulin B chain by wild-type NEP was analyzed by reverse-phase HPLC following the appearance of each product as a function of time, **Figure 1**. Product peaks were collected and subsequently analyzed by mass spectrometry to determine their identities. As shown in **Table 4** and the insert in **Figure 1** this analysis identified seven cleavage sites. Based on the order of appearance of each peak, it is likely that insulin B chain_1–10_, insulin B chain_1–11_, insulin B chain_1–14_ and its partner peak insulin B chain_15–30_, insulin B chain_17–30_, and insulin B chain_24–30_ are all products of primary cleavages. These products appear at the first time point when approximately 15% hydrolysis of insulin B chain had occurred (30 minutes). At 30% hydrolysis (90 min.), peaks corresponding to insulin B chain_1–5_ and insulin B chain_17–24_ are observed. Peaks for insulin B chain_1–16_ and insulin B chain_15–23_ become apparent at >30% insulin B chain hydrolysis. It should be noted that the expected product peaks insulin B chain_11–30_ and insulin B chain_12–30_, the partner products of insulin B chain_1–10_ and insulin B chain_1–11_ respectively, were identified by mass spectrometry, however the peaks never accumulated significantly above the baseline throughout the incubation. It is likely that these are transient products that are subsequently cleaved contributing to other product peaks (i.e. insulin B chain_24–30_).

**Table 4:** Products of insulin B chain hydrolysis by NEP.

Retention Time				
(min.)	Insulin B Chain fragment	Expected Mass	Observed Mass	Cleavage Site
9.1	1–5	643.31	643.24	H^5^-L^6^
18.3	1–10	1188.48	1188.39	H^10^-L^11^
22.7	17–24	927.42	927.35	Y^16^-L^17^+F^24^-F^25^
24.3	1–11	1301.6	1301.47	L^11^-V^12^
24.7	15–23	1056.51	1056.42	A^14^-L^15^+G^23^-F^24^
25.4	1–14	1600.75	1600.64	A^14^-L^15^
26.2	24–30	872.44	872.39	G^23^-F^24^
29.1	17–30	1634.79	1634.64	Y^16^-L^17^
31.9	1–16	1876.89	1876.75	Y^16^-L^17^
33.3	15–30	1910.93	1910.8	A^14^-L^15^
33.6	12–30	2210.87	2209.93	L^11^-V^12^
35.5	11–30	2323.17	2323.06	H^10^-L^11^
36.6	1–30	3493.67	3493.52	(insulin B chain)

NEP mediated hydrolysis was carried out as described in **Table 2**. The reaction was stopped by adding 10 µL of 5% TFA when approximately half of the substrate had been hydrolyzed (180 min.). The acidified reaction mixture was subjected to HPLC as described in figure 1, individual peaks were collected and identified by mass spectral analysis.

### Analysis of NEP mutant cleavage of insulin B chain

**Figure 2:**
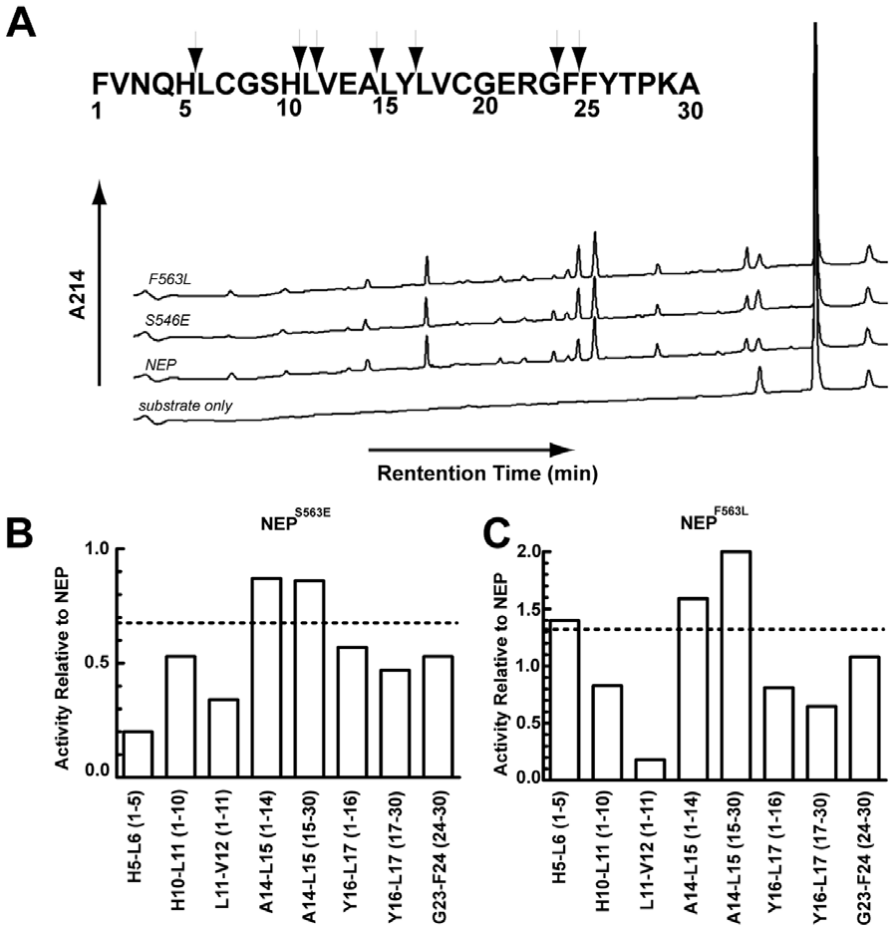
Comparison of insulin B chain cleavage between NEP, NEP^F563L^, and NEP^S546E^. A. HPLC profile of insulin B chain cleaved by NEP and NEP mutants at 30% hydrolysis. B. Rates of peak accumulation at each cleavage site normalized to that of NEP for mutant NEP^S546E^. C. Rates of peak accumulation at each cleavage site normalized to that of NEP for mutant NEP^F563L^. Dotted lines indicate the overall rate of hydrolysis of insulin B chain from **Table 2**. Reactions were carried out at 37°C with 15 µM insulin B chain in 20 mM MES, pH 6.5.

As a representative of the NEP^F563^ and NEP^S546^ mutants, we compared the cleavage profile of NEP^F563L^ and NEP^S546E^ to that of NEP using time course experiments, **Figure 2**. By adjusting the amount of NEP mutant used, the rate of hydrolysis of insulin B chain by all NEP forms was virtually identical. The overall cleavage profile at 30% substrate hydrolysis revealed that all of the product peaks observed with NEP are present with the mutant enzymes indicating that there were no unique or missing cleavage sites between NEP^F563L^, NEP^S546E^ and NEP, **Figure 2A**. Since rates were based on peak areas measured at 214 nm, which in turn is dependent on both the number of peptide bonds and the number of aromatic residues within a given peptide, only the observed rates of change for a particular peptide product can be compared between enzyme forms. A comparison of the rate of change of different peaks within the same enzyme form or between enzyme forms is not valid under our conditions of analysis.

### NEP mutations affect cleavage sites preferences in insulin B chain

**Table 5** and **Figure 2B,C** show the rates of product accumulation normalized to the amount of NEP protein present. Relative to wild-type NEP the overall rate of hydrolysis of insulin B chain is slightly increased in NEP^F563L^ and slightly decreased in NEP^S546E^, **Table 2**. Thus one scenario is that all sites in insulin B chain would be cleaved at the same relative rate compared to wild-type NEP. Alternatively, the introduced mutations may differentially affect specific cleavage sites. The data in **Table 5** clearly shows the latter scenario with differential effects of mutations on specific cleavages. NEP^S546E^ cleaves insulin B chain at an overall rate 0.7 times that of NEP, however it is clear that cleavage at A^14^-L^15^ is nearly identical between NEP and this mutant. Cleavage at H^5^-L^6^ is well below the overall insulin B chain rate of 0.7 (**Figure 2B**), while cleavages at H^10^-L^11^, Y^16^-L^17^, and G^23^-F^24^ are all slightly slower than the expected 0.7 times the wild-type rate.

**Table 5:** Relative rates of accumulation of peaks generated by the NEP and NEP mutant dependent hydrolysis of insulin B chain.

Single Cleavage				
	Peak	NEP	S546E	F563L
Cleavage Site		Δarea/min/ng	Δarea/min/ng	Δarea/min/ng
H^5^-L^6^	1–5	59	12	83
H^10^-L^11^	1–10	163	87	135
L^11^-V^12^	1–11	88	30	16
A^14^-L^15^	1–14	167	145	266
	15–30	135	116	282
Y^16^-L^17^	1–16	31	17	25
	17–30	171	81	112
G^23^-F^24^	24–30	268	142	290

Two Cleavages				
	Peak	NEP	S546E	F563L
Cleavage Sites		Δarea/min/ng	Δarea/min/ng	Δarea/min/ng
A^14^-L^15^, G^23^-F^24^	15–23	23	26	64
Y^16^-L^17^, F^24^-F^25^	17–24	33	18	25

Time course assays were carried out by incubation of NEP with insulin B chain using conditions as described in **Table 2**. At 0, 30, 60, 90, 120, and 180 min., aliquots of 100 µL were removed followed by the addition of 10 µL of 5% TFA to stop further hydrolysis. Each reaction mixture was subjected to HPLC analysis as in **Table 4** and peak areas measured. The rate of accumulation for each peak was calculated from the linear phase of the reaction.

NEP^F563L^ cleaves insulin B chain at a rate 1.4 times that of NEP. Similar to that seen with NEP^S546E^, NEP^F563L^ products produced from single cleavage sites exhibit noticeably different rates compared to NEP, **Figure 2C**. Cleavage at A^14^-L^15^, H^5^-L^6^, and G^23^-F^24^ are close to the expected 1.4 times faster that of NEP, but cleavage at H^10^-L^11^, L^11^-V^12^, and Y^16^-L^17^ are slower than NEP, (0.8 times, 0.2 times, and ∼0.7 times the NEP rate respectively, rather than the overall 1.4 times faster than the NEP rate).

Based on the data in **Table 5** the elevated activity of NEP^F563L^ towards insulin B chain can likely be attributed to an increased rate of cleavage at the primary cleavage site A^14^-L^15^. Although this cleavage involves a leucine residue, the finding that cleavage at Y^16^-L^17^ is slower than with wild-type NEP shows the enhanced cleavage at A^14^-L^15^ is not due to simply the F563L mutation producing enhanced reactivity toward leucine, but more likely an effect of neighboring residues.

### Identification of NEP cleavage sites in Aß_1–40_

**Figure 3:**
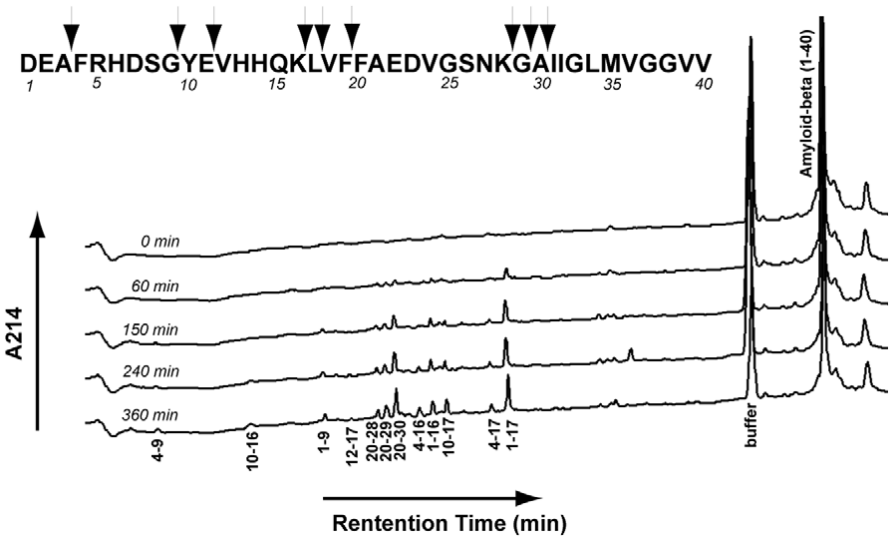
Time course of NEP mediated hydrolysis of Aß_1–40_. Time course measurements were carried out by incubation of NEP with 24 µM Aß_1–40_ in 20 mM MES, pH 6.5, at 37°. 100 µL aliquots were removed at each time point and 10 µL of 5% TFA was added to stop the reaction. Samples were analyzed as described in **Figure 1**. Numbers under each peak indicate the identification of the peptide by sequence.

To further study the effect of mutations on cleavage site specificity, time course assays were also performed for the hydrolysis of the physiological substrate Aß_1–40_. Like the analysis of insulin B chain, a time course assay was first done with NEP to identify cleavage products, **Figure 3** **and** **Table 6**. After 15% hydrolysis of Aß_1–40_ (150 min.) by NEP there were six discernable product peaks corresponding to Aß_1–16_, Aß_1–17_, Aß_10–17_, Aß_20–28_, Aß_20–29_, and Aß_20–30_. At 25% hydrolysis (240 min.), peaks corresponding to Aß_1–9_, Aß_4–16_, and Aß_4–17_ were observed, while at 40% hydrolysis peaks Aß_4–9_ and Aß_10–16_ appeared. Peak Aß_12–17_ was the last peak to be observed at 45% hydrolysis of Aß_1–40_ (360 min.). Missing from the HPLC analysis were the C-terminal products resulting from the cleavages at K^28^-G^29^, G^29^-A^30^, and A^30^-I^31^. These products are derived from the trans-membrane region of the amyloid precursor protein (APP) from which Aß is formed and are rather hydrophobic. It is likely that these peptides were not eluted in the gradient we employed.

**Table 6:** Identification of products resulting from the hydrolysis of Aß_1–40_ by NEP.

Retention Time (min.)	Amyloid ß peptide fragment	Expected Mass	Observed Mass	Cleavage Site
7.4	4–9	717.32	717.30	E^3^-F^4^+G^9^-Y^10^
10.3	10–16	939.46	939.44	G^9^-Y^10^+K^16^-L^17^
14.7	1–9	1032.43	1032.43	G^9^-Y^10^
15.8	12–17	760.43	760.44	E^11^-V^12^+L^17^-V^18^
17.2	20–28	964.45	965.46	F^19^-F^20^+K^28^-G^29^
17.4	20–29	1022.44	1022.47	F^19^-F^20^+V^29^-A^30^
18.2	20–30	1093.50	1093.52	F^19^-F^20^+A^30^+I^31^
19.0	4–16	1638.76	1638.76	E^3^-F^4^+K^16^-L^17^
19.8	1–16	1953.87	1953.83	K^16^-L^17^
20.4	10–17	1052.54	1052.54	G^9^-Y^10^+L^17^-V^18^
22.3	4–17	1751.85	1751.81	E^3^-F^4^+L^17^-V^18^
23.1	1–17	2066.96	2066.92	L^17^-V^18^
37.4	1–40	4327.00	4327.15	(Ab_1–40_)

NEP mediated hydrolysis was carried out as described in **Table 2**. The reaction was stopped by adding 10 µL of 5% TFA when approximately half of the substrate had been hydrolyzed (360 min.). The acidified reaction mixture was subjected to HPLC analysis as described in **Table 4**. Each peak was isolated and subjected to mass spectral analysis.

Two other studies have identified NEP cleavage sites within Aß_1–40_ [25], [26]. These studies as well as the current study all detected cleavages at G^9^-Y^10^, F^19^-F^20^, and A^30^-I^31^. Cleavage at A^3^-F^4^ was detected in this study as well as by Howell et al [25], [26]. Cleavages at K^28^-G^29^ and G^29^-A^30^ were detected in this study as well as by Leissring et al. [25], [26]. In the current study additional unreported cleavage sites at E^11^-V^12^, K^16^-L^17^, and L^17^-V^18^ were detected. These were previously pointed out as potential cleavage sites, but were not observed [25], [26]. Both Leissring and Howell identified cleavage at G33-L34, yet this cleavage was not found in this study. These differences likely reflect differences in the resolution of the Aß_1–40_ cleavage products on the HPLC columns used and gradient conditions.

### Analysis of specific cleavage sites in Aß_1–40_

**Figure 4:**
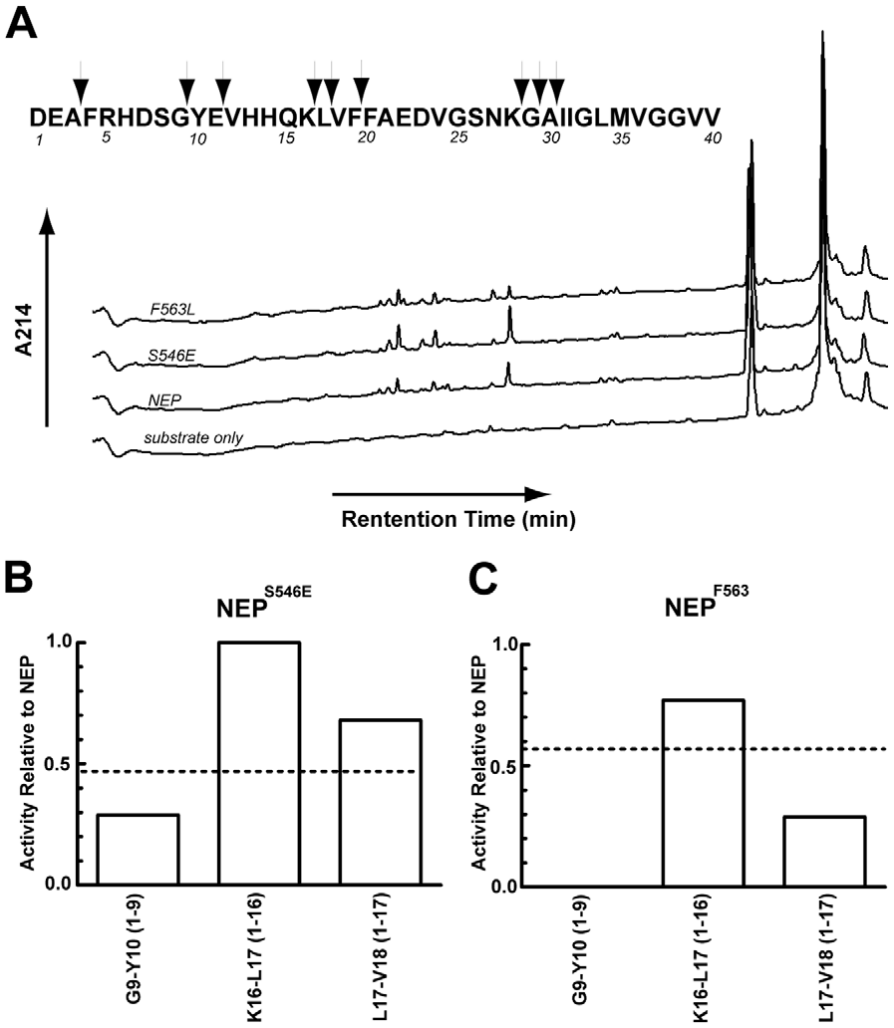
Comparison of Aß_1–40_ cleavage between NEP, NEP^F563L^, and NEP^S546E^. A. HPLC cleavage profile of Aß_1–40_ cleavage by NEP and NEP mutants at ∼30% hydrolysis. B. Rates of peak accumulation at each cleavage site normalized to that of NEP for mutant NEP^S546E^. C. Rates of peak accumulation at each cleavage site normalized to that of NEP for mutant NEP^F563L^. Dotted lines indicate the overall rate of hydrolysis of Aß_1–40_ from **Table 2**. Reactions were carried out at 37°C with 15 µM Aß_1–40_ in 20 mM MES, pH 6.5.

We next compared the Aß_1–40_ cleavage profile of the NEP^F563L^ and NEP^S546E^ mutants to that of wild-type NEP, **Figure 4**. Since NEP, NEP^F563L^ and NEP^S546E^ cleave Aß_1–40_ at different rates, the amount of the mutant enzymes used in the reaction was as before adjusted in order to analyze products formed at the same fraction of degradation. Interestingly, although all wild-type peaks were present in the NEP^S563E^ mutant, the peak corresponding to Aß_1–9_ was not present among the hydrolysis products of the NEP^F563L^ mutant.

Similar to the analysis conducted with insulin B chain, the linear rate of product accumulation was determined for each peak and normalized to the amount of protein in the reaction, **Table 7**. Unlike the hydrolysis of insulin B chain, the hydrolysis of Aß_1–40_, even at the earliest time points, produced products resulting from multiple cleavage events. Out of the 12 identified product peaks, only 3 could be produced by a single cleavage. Rates for all cleavages were calculated and are given in **Figure 4, B–D**, but only the three putative primary cleavage sites can be compared.

**Table 7:** Accumulation rates of products of NEP dependent cleavage of Aß_1–40_.

Single Cleavage				
	Peak	NEP	S546E	F563L
Cleavage Site		Δarea/min/ng	Δarea/min/ng	Δarea/min/ng
G^9^-Y^10^	1–9	7	2	0
K^16^-L^17^	1–16	22	22	17
L^17^-V^18^	1–17	75	51	22

Dual Cleavages				
	Peak	NEP	S546E	F563L
Cleavage Sites		Δarea/min/ng	Δarea/min/ng	Δarea/min/ng
A^3^-F^4^, G^9^-Y^10^	4–9	10	4	5
A^3^-F^4^, K^16^-L^17^	4–16	7	5	5
A^3^-F^4^, L^17^-V^18^	4–17	9	3	12
G^9^-Y^10^, K^16^-L^17^	10–16	4	5	11
G^9^-Y^10^, L^17^-V^18^	10–17	18	5	8
E^11^-V^12^, L^17^-V^18^	12–17	3	2	1
F^19^-F^20^, K^28^-G^29^	20–28	11	2	5
F^19^-F^20^, G^29^-A^30^	20–29	16	5	6
F^19^-F^20^, A^30^-I^31^	20–30	34	15	26

Time course assays were carried out by incubation of NEP with 24 µM Aß_1–40_ using reaction conditions as described in **Table 5**. At 0, 60, 150, 240, and 360 min., aliquots of 100 µL were removed followed by the addition of 10 µL of 5% TFA to stop further hydrolysis. Each reaction mixture was subjected to HPLC analysis as in **Table 5** and peak areas measured. The rate of accumulation for each peak was calculated from the linear phase of the reaction.

NEP^S546E^ hydrolyzes Aß_1–40_ at an overall rate 0.5 times that of wild-type NEP. Two of the three products resulting from a possible single cleavage by NEP^S546E^ exhibit a higher than predicted rate of cleavage. Cleavage at K^16^-L^17^ producing Aß_1–16_ is identical rather than half the wild-type rate while cleavage at L^17^-V^18^ producing Aß_1–17_ was approximately 70% rather than 50% of the NEP rate. The remaining single cleavage site at G^9^-Y^10^ shows a slower rate being about 30% that of wild-type enzyme. Thus it would appear that all three of these cleavages contribute to the overall rate and together produce an average rate 0.5 times that of NEP.

Cleavage at L^17^-V^18^ is approximately three times faster for NEP^S546E^ compared to NEP^F563L^. Thus cleavage at L^17^-V^18^ for mutant NEP^S563E^ is 70% of the wild-type NEP whereas cleavage at this bond for the NEP^F563L^ is 25% of NEP. In order to account for this finding there must either be an undetected cleavage that is significantly reduced in NEP^S546E^ or more likely that NEP^F563L^ exhibits a unique cleavage pattern that yields these products.

The rate of appearance of Aß_1–16_ is linear over the entire 360-minute time course for all three enzymes. Aß_1–17_, on the other hand, shows a linear increase with both NEP^F563L^ and NEP^S546E^, but is non-linear with wild-type NEP showing very little increase after 150 min. This is consistent with Aß_1–17_ being further metabolized by NEP, most likely by being cleaved at G^9^-Y^10^ giving rise to Aß_1–9_ and Aß_10–17_, both of which show higher peak areas in NEP than with either mutant. In contrast the absence of an obvious reduction of Aß_1–9_ and Aß_10–17_ in the reaction of NEP^F563L^ and NEP^S546E^ suggests these mutants cleave the G^9^-Y^10^ bond at a much slower rate.

Although NEP^F563L^ did not produce a discrete Aß_1–9_ peak, it appears to cleave the G^9^-Y^10^ bond as evidenced by the presence of the products Aß_10–16_ and Aß_10–17_. Since Aß_1–9_ is absent in the NEP^F563L^ profile but Aß_10–16_ and Aß_10–17_ are observed, the cleavage by NEP^F563L^ at G^9^-Y^10^ is likely dependent on the cleavage at A^3^-F^4^. Aß_1–9_ is not further degraded by hydrolysis at the A^3^-F^4^ site with both NEP and NEP^S546E^ as evidenced by its linear increase as a function of time. If hydrolysis occurred at the A^3^-F^4^ bond of the Aß_1–9_ product, one would expect either no time dependent increase or a decrease in the Aß_1–9_ peak.

The finding that most of the observed products of Aß_1–40_ cleavage result from multiple cleavages, even for early time points, would suggest that NEP is processive in its cleavage of Aß_1–40_ and can make several cleavages before product release. Whether the enzyme does this with both C-terminal and N-terminal products is not clear and how the products are reoriented in the active site for additional cleavages is also unclear. However, the alternative explanation for observing products derived from multiple cleavages requires an extremely high affinity of the product peptide to be bound and cleaved in the presence of a large amount of unreached Aß_1–40_. The processive model is consistent with the overall structure of the enzyme, which has only a small opening leading to the large, enclosed chamber that borders the active site. Once a peptide diffuses through the narrow opening, it is likely that it and product peptides are retained in the enclosed chamber sufficiently long enough for multiple active site binding events to occur.

Of the eleven substitutions made at Phe^563^ only four produced enzyme of sufficient stability to be studied. The four residues that did produce stable forms of NEP all represented conservative change to hydrophobic residues, whereas the other non-conservative or semi-conservative changes produced unstable enzyme forms. This suggests position 563 likely serves as an important anchor residue in the folding of the enzyme, and interaction of a hydrophobic residue at this position with other hydrophobic and aromatic residues is required. Changes at Ser^546^ produced more variable results in terms of enzyme expression, and this position is therefore likely less critical in folding.

Amino acid substitutions at both Phe^563^ and Ser^546^ affected the cleavage pattern of NEP. Phe^563^ forms part of the S1′ substrate binding pocket and helps define the specificity of NEP for hydrophobic/aromatic P1′ residues, thus changing this amino acid would likely affect cleavage specificity. Ser^546^ is positioned to contribute to the S2/S3 binding site, although this registration is more speculative, since subsites N terminal to the scissile bond are not defined by available structures with bound inhibitors. Although selectivity is less stringent at these positions, we have previously obtained evidence that residues N terminal to the scissile bond, particularly the S1 subsite, also contribute to substrate specificity [27] and this study certainly supports a role for the P2/P3 peptide positions in selectivity.

Of the two mutants studied in detail no changes in the peptide bonds that were cleaved were observed, but the relative rates of cleavage were affected by substitution at both Phe^563^ and Ser^546^. In general substitution of leucine for phenylalanine at the S′1 site either had no effect or increased the rate at which cleavage occurred with a P′1 leucine or phenylalanine residue, but significantly decreased the rate when valine occupied the P′1 position. Since the NEP^F563L^ mutation substitutes a smaller residue in the hydrophobic S1′ subsite, this result can be rationalized on the basis that the relatively small valine at P1′ leaves an unfavorable gap upon substrate binding. Substitution of glutamate for serine at the S2/S3 position produced marked differential changes in cleavage rates, but these changes were complex and not easily rationalized. This result extends our earlier results indicating that residues N terminal to the scissile bond play an important role in selectivity. It is possible given the complex nature of the observed rate changes that positioning of the N terminal side of the substrate peptide may vary in a sequence dependent manner.

Taken together this study shows that a single amino acid substitution within the active site of NEP can cause changes in cleavage site preference, which strongly supports the notion that it may be possible to alter the NEP active site to generate substrate specific variants that will be useful therapeutically.

## Materials and Methods

### Mutagenesis and production of expression vectors

NEP variants were constructed as gene segment cassette modules using degenerative oligonucleotide primers to introduce sequence diversity by PCR. Individual mutation cassettes were inserted into the pCDNA-shNEP-CHis (SacII+Pst1) expression vector, a re-engineered pCDNA-3.1 vector with a silent SacII mutation introduced 5′ to the active site region within a secreted form of the human NEP (shNEP) coding sequence and a C-terminal hexahistidine affinity tag. Two silent mutations were made, using the Quickchange® II site directed mutagenesis kit (Stratagene) to eliminate additional PstI sites and facilitate cassette subcloning of the shNEP gene. A 3 kb fragment of lambda “stuffer” DNA was inserted between the SacII and Pst1 sites to allow gene segment cassette subcloning while eliminating wild-type sequences from being selected [27], [28]. Non-polar substitutions at Phe^563^ were initially cloned into and sequenced using the pBPG1 vector for expression in yeast [29]. However, low enzyme yields from yeast led to the removal of the identified clones from the pBPG1 vector and subcloning into the pDNA-shNEP-CHis-(SacII-3kbstuffer-PstI) vector. PCR based mutagenesis was used to create NEP variants using degenerative primers containing appropriate restriction sites. For making polar substitutions at Phe^563^ the 5′ primer contained a PstI restriction site and the 3′ primer contained an NcoI restriction site to facilitate movement from the pBPG1 vector to the pCDNA-shNEP-CHis- (SacII-3kbstuffer-PstI) vector. The degenerative primers (IDTDNA, Corralville, IA) used were:

5′-gattcggcttgtacagcatatgtgg-3′ (S546 reverse primer)

5′-gggggctgcagaatgccggctgggaagactatctgatttcttcctgaTBYgtaaaatgcattgactaccgcg-3′ (S546 forward primer)

5′-cccattctgcagccccccVtStttagtgcccagcagtccaac-3′ (F563 forward primer for non polar residues)

5′-cccattctgcagccccccVDStttagtgcccagcagtccaac-3′ (F563 forward primer for polar residues)

5′-tccaccagtcaacgaggtctc-3′ (F563 reverse primer)

where V = A, C, or G; S = C or G; D = A, G, or T; B = C, G, or T; Y = C or T.

### Expression and purification

NEP protein was expressed in HEK293T cells transfected with the pcDNA vector described above. Cells were grown in Dulbecco's Modified Eagle Medium (DMEM, Gibco) containing 10% FBS and 44 mM NaHCO_3_ added as a supplement. For transfections, Polyfector (BamaGen Bioscience) and plasmid DNA were incubated at room temperature for 20 min in serum free DMEM media and then added to HEK293T cells in the DMEM media. The media was replaced with serum free DMEM 12–14 hours post transfection, and collected 72–96 hours post transfection. To the media was added 1 M Tris-HCl, pH 7.4 to a final concentration of 50 mM and the secreted enzyme was then purified on a His-Select Affinity Agarose Column (Sigma). The affinity purification step yielded enzyme with the purity dependent on the level of NEP expression. Activities measured in this study were attributed to NEP, since a mock transfection and purification resulted in no activity toward any of the substrates tested and NEP inhibitors eliminated all activity. We estimated the amount of NEP protein present by running the purified preparations on 8% or 10% SDS-PAGE gels along with purified NEP as a standard. The gels were stained with Sypro Ruby dye scanned on a Typhoon 9400 Imager, and quantified with Image Quant 5.2 software. In preliminary experiments the gel was transferred to a polyvinylidene fluoride (PVDF) and subject to Western blot analysis using goat anti-mNEP at 1∶1000 (R&D systems) as the primary antibody and anti-goat IRDye800 at 1∶20,000 (Rockland) as the secondary antibody. Probed membranes were imaged using an Odyssey infrared imager and Odyssey 2.1 software. Intensities of each band were analyzed with Image Quant 5.2 software. Data was analyzed using Prism4 software.

### Activity assays

NEP activity was routinely assayed using the fluorogenic peptide glutaryl-Ala-Ala-Phe-4-methoxy-2-naphthylamide (Glut-Ala-Ala-Phe-MNA, Sigma) [30]. Reactions of 400 µl contained 100 µM Glut-Ala-Ala-Phe-MNA, 1 µg of aminopeptidase [31] and 15 to 100 ng of NEP or mutant NEP depending on their activity in 20 mM MES buffer, pH 6.5. Activity was monitored with a Spectra Max Gemini XS plate reader using an excitation wavelength of 340 nm and an emission wavelength of 425 nm. Reaction specificity was determined using the NEP inhibitors phosphoramidon and CGS 24592 [24], the latter being a highly specific and potent inhibitor.

### Kinetic Analysis

Kinetic constants for NEP and its mutants were obtained using the assay conditions noted above, but with Glut-Ala-Ala-Phe-MNA varied from 20 to 500 µM. Typically 12 data points were obtained. The data were fit to the Michaelis-Menten equation using Prism4 software. The K_i_ for insulin B chain was obtained by measuring the rate of Glut-Ala-Ala-Phe-MNA hydrolysis in the presence of varying concentrations of insulin B chain from 1 to 40 µM. Data were fit to a Dixon plot (1/rate versus [insulin B chain]) using Prism4 software and the ID_50_ obtained as the –x intercept, where ID_50_ corresponds to the concentration of insulin producing 50% inhibition. The actual Ki was obtained from the equation: ID_50_ = K_i_ (1+ [Glut-Ala-Ala-Phe-MNA]/K_mGlut-Ala-Ala-Phe-MNA_) [32], [33].

### HPLC assays

Cleavage of physiological peptides was measured via reverse phase high performance liquid chromatography (HPLC) following incubation of the purified NEP or its mutants with 15 µM insulin B chain (Sigma Aldrich), 24 µM Aß_1–40_ (Anaspec), or 64 µM leu-ENK (Sigma) in 100 µL of 20 mM MES, pH 6.5, at 37°C. Reactions were run in triplicate. HPLC was carried out in a Vydac C4 column using a linear gradient from 0.1% trifluoroacetic acid (TFA) in 95% water, 5% acetonitrile to 0.1% TFA in 50% acetonitrile/water at a flow rate of 1 mL/min. Peptides and hydrolysis products were detected at 214 nm and quantified by measuring peak areas. Peptides obtained from HPLC were analyzed on an Applied Biosystems 4800 MALDI TOF/TOF Proteomics Analyzer at the University of Kentucky Proteomics core. This facility is supported in part by grant P20RR020171 from the NIH/NCRR.

### Synthesis and analysis of NEP cDNA

In order to compare NEP transcript levels, RNA from 96-hour post-transfected HEK cells was collected using a QIAshredder column (Qiagen) and an RNeasy Mini Kit (Qiagen). Using 5 µg of the harvested RNA, cDNAs were produced with a Superscript First Strand Synthesis kit (Invitrogen) using the oligo(dT) primer included in the kit. Using NEP specific primers (5′-aaagtaaacaactgaaga-3 and 5′-tcctgaaattgcctggac-3′) and primers for β-actin (5′-taggagccagagcagtaatc-3′ and 5′-tgtttgagaccttcaacacc-3′) for controls, relative levels of cDNA were measured by comparing product formation at 20, 25, 30, and 35 cycles in PCR reaction comparing under standard conditions using 50°C annealing temperature.

### Statistical Analysis

Statistical analysis comparing wild-type NEP and its mutants was performed with Prism 4 software using a two-tailed paired t-test with a 95% confidence interval.

## Supporting Information

Figure S1 **Sites Mutated in NEP.** The active site region of the NEP-phosphoramidon complex [23] is shown with the protein in a ribbon and surface representation and the bound ligand in a stick representation. The mutated residue positions are in red with side chains shown. The zinc ion cofactor is represented by a yellow sphere. Phosphoramidon residues equivalent to substrate peptide positions P1–P2′ are indicated. The approximate position of substrate P2 and P3 residues is shown by the blue ovals. Purple arcs indicate contact between the P1′ residue and F563.
(TIF)

Figure S2 **NEP and mutant NEPs produce similar levels of mRNA.** Varying PCR cycles were used to estimate the relative amount of NEP mRNA of high and low expressing mutants. Total RNA was harvested from HEK293T cells 96 hrs post transfection and an equal amount of RNA was used as a template for first-strand synthesis to produce a cDNA library using an oligo(dT) universal primer. The cDNA libraries were then used as templates for PCR using primers specific for NEP (experimental) and b-Actin (control). Samples from PCR cycles 20, 25, and 30 were used to estimate NEP transcript levels. The NEP^F563K^ mutant product band intensity relative to NEP was 0.9, 1.0, and 1.3 at cycles 20, 25, and 30 respectively. NEP^F563V^ and NEP^F563L^ were at a level approximately half of the wild-type NEP transcript. In contrast, NEP^F563L^ exhibited the same activity as wild-type enzyme while NEP^F563V^ displayed ∼25% of the wild-type activity, while the activity for NEP^F563K^ was undetectable (<1% relative to wild-type enzyme) under our assay conditions (**Table 1**).
(TIF)

Figure S3 **Determination of the concentration of NEP mutants.** Purified NEP samples were subjected to SDS-PAGE on 8% polyacrylamide gels and stained for protein with Sypro Ruby dye (A). The gel contained 100, 250, and 500 ng of purified NEP, which was used to construct a standard curve (C) from which the concentration of each NEP form was calculated. Samples of purified NEP, NEP^F563L^, and NEP^S536E^ were run at 15 µl and 30 µl. Intensities of each NEP band were fit to the standard curve (C) to give 8.9±0, 12.7±3.7, and 11.6±1.1 ng/µL for NEP, NEP^F563L^, and NEP^S536E^, respectively (E, solid bars). Similarly a Western blot derived from a 10% SDS-PAGE was run containing 10, 50, and 100 ng of purified NEP from which a standard curve was derived (D). NEP, NEP^F563L^, and NEP^S536E^ were run at 3.75 µl and 7.50 µl. Intensities of each NEP band were fit to the standard curve (D) to give 11.0±1.4, 13.3±0.6, and 13.1±2.2 ng/µL for NEP, NEP^F563L^, and NEP^S546E^, respectively (E, empty bars). Note - the difference in size between the NEP standard and the NEP experimental samples is due to differences in glycosylation between NEP isolated from CHO cells and HEK cells, respectively.
(TIF)
