# Supplementary figures and images for: Active Site Mutations Change the Cleavage Specificity of Neprilysin
Source: PLoS One. 2012 Feb 23;7(2):e32343. doi: 10.1371/journal.pone.0032343 (PMC3285688; doi:10.1371/journal.pone.0032343)

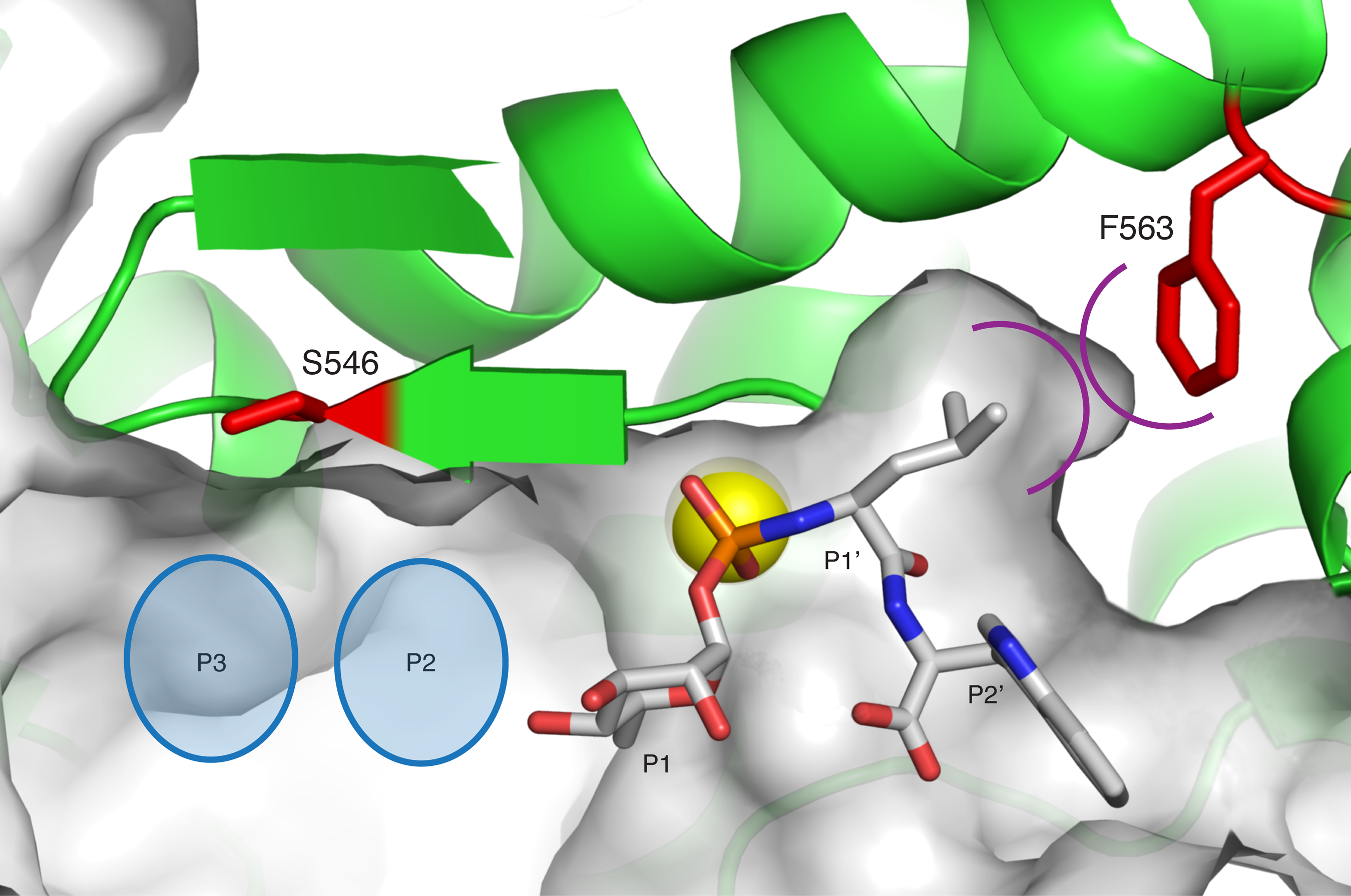

Supplement: Figure S1 — Sites Mutated in NEP. The active site region of the NEP-phosphoramidon complex [23] is shown with the protein in a ribbon and surface representation and the bound ligand in a stick representation. The mutated residue positions are in red with side chains shown. The zinc ion cofactor is represented by a yellow sphere. Phosphoramidon residues equivalent to substrate peptide positions P1–P2′ are indicated. The approximate position of substrate P2 and P3 residues is shown by the blue ovals. Purple arcs indicate contact between the P1′ residue and F563. (TIF) [file pone.0032343.s001.tif]

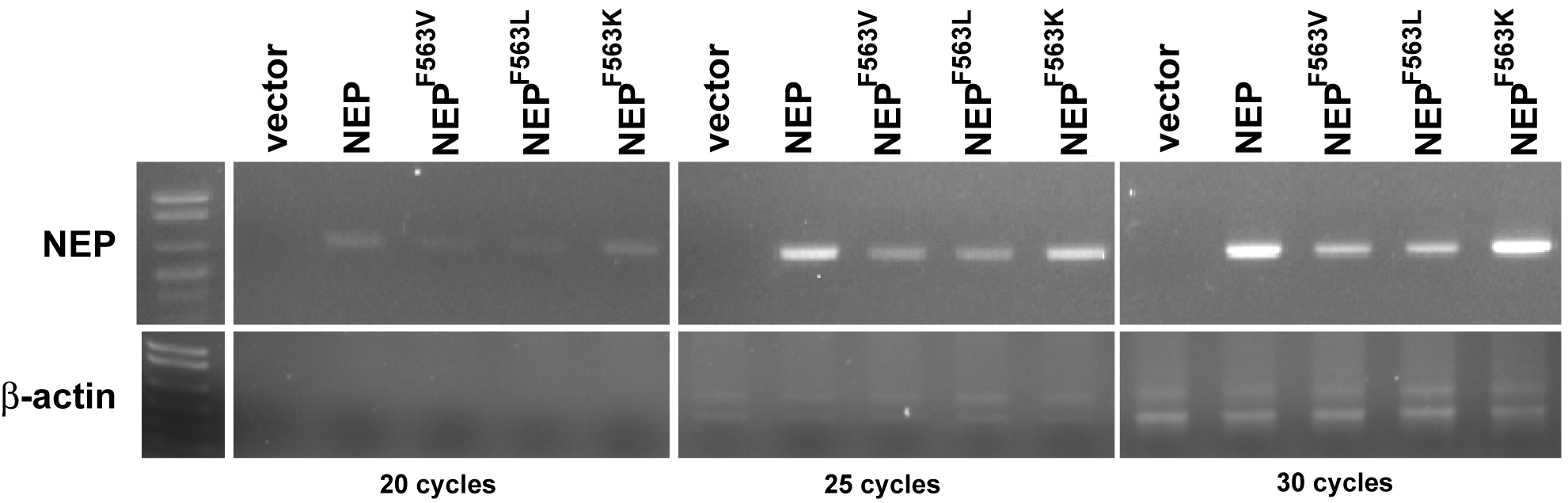

Supplement: Figure S2 — NEP and mutant NEPs produce similar levels of mRNA. Varying PCR cycles were used to estimate the relative amount of NEP mRNA of high and low expressing mutants. Total RNA was harvested from HEK293T cells 96 hrs post transfection and an equal amount of RNA was used as a template for first-strand synthesis to produce a cDNA library using an oligo(dT) universal primer. The cDNA libraries were then used as templates for PCR using primers specific for NEP (experimental) and b-Actin (control). Samples from PCR cycles 20, 25, and 30 were used to estimate NEP transcript levels. The NEPF563K mutant product band intensity relative to NEP was 0.9, 1.0, and 1.3 at cycles 20, 25, and 30 respectively. NEPF563V and NEPF563L were at a level approximately half of the wild-type NEP transcript. In contrast, NEPF563L exhibited the same activity as wild-type enzyme while NEPF563V displayed ∼25% of the wild-type activity, while the activity for NEPF563K was undetectable (<1% relative to wild-type enzyme) under our assay conditions ( Table 1 ). (TIF) [file pone.0032343.s002.tif]

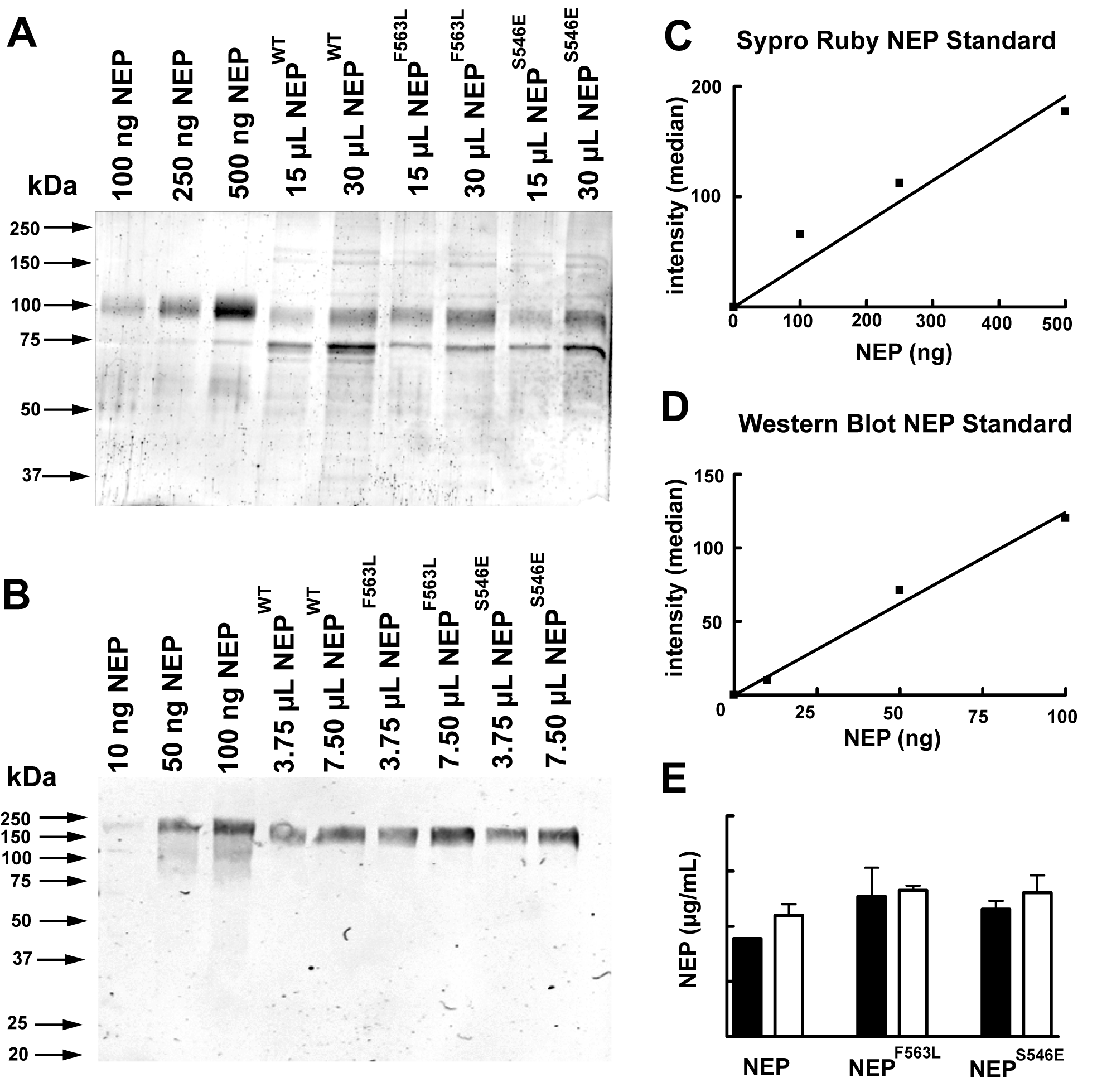

Supplement: Figure S3 — Determination of the concentration of NEP mutants. Purified NEP samples were subjected to SDS-PAGE on 8% polyacrylamide gels and stained for protein with Sypro Ruby dye (A). The gel contained 100, 250, and 500 ng of purified NEP, which was used to construct a standard curve (C) from which the concentration of each NEP form was calculated. Samples of purified NEP, NEPF563L, and NEPS536E were run at 15 µl and 30 µl. Intensities of each NEP band were fit to the standard curve (C) to give 8.9±0, 12.7±3.7, and 11.6±1.1 ng/µL for NEP, NEPF563L, and NEPS536E, respectively (E, solid bars). Similarly a Western blot derived from a 10% SDS-PAGE was run containing 10, 50, and 100 ng of purified NEP from which a standard curve was derived (D). NEP, NEPF563L, and NEPS536E were run at 3.75 µl and 7.50 µl. Intensities of each NEP band were fit to the standard curve (D) to give 11.0±1.4, 13.3±0.6, and 13.1±2.2 ng/µL for NEP, NEPF563L, and NEPS546E, respectively (E, empty bars). Note - the difference in size between the NEP standard and the NEP experimental samples is due to differences in glycosylation between NEP isolated from CHO cells and HEK cells, respectively. (TIF) [file pone.0032343.s003.tif]
